# Causal Associations Between Dietary Factors and Uterine Inflammatory Diseases: A Mendelian Randomization Study

**DOI:** 10.1002/fsn3.71076

**Published:** 2025-10-21

**Authors:** Biao Xiong, Zhaoan Lian

**Affiliations:** ^1^ Department of Obstetrics and Gynecology The Central Hospital of Enshi Tujia and Miao Autonomous Prefecture Enshi Hubei China

**Keywords:** dietary factors, mendelian randomization, uterine inflammatory diseases, womens health

## Abstract

Uterine inflammatory diseases, such as endometritis, are linked to infertility and adverse reproductive outcomes. We performed a two‐sample Mendelian randomization (MR) study using genetic instruments for 19 dietary exposures (e.g., fruits, meats, cereals, beverages) from UK Biobank GWAS. The outcome was inflammatory uterine disease (ICD‐10: N71) from FinnGen. We applied inverse‐variance weighted (IVW) regression as the primary analysis, with sensitivity analyses (MR‐Egger, weighted median) and pleiotropy‐robust methods (MR‐PRESSO). Higher dried fruit intake showed a robust inverse association with uterine inflammatory disease (IVW OR = 0.277, 95% CI: 0.154–0.498; *p* = 1.72 × 10^−5^, FDR‐q = 3.27 × 10^−4^), supported by weighted median analysis. Cereal intake was nominally protective (IVW OR = 0.446, 95% CI: 0.255–0.783; *p* = 0.00488, FDR‐q = 0.0464) but not significant in weighted median analyses. No other dietary factors (alcohol, meat, fresh fruits) reached significance. MR‐Egger detected no pleiotropy (*p* > 0.05). This study predicted dried fruit consumption and cereal consumption may reduce uterine inflammatory disease risk, suggesting diet as a modifiable protective factor. Further studies should validate mechanisms.

## Graphical abstract

This Mendelian randomization study investigated causal links between 19 dietary factors and uterine inflammatory diseases. Higher dried fruit intake showed a robust inverse association with reduced risk (IVW OR = 0.277, *p* = 1.72 × 10–5), while cereal intake was nominally protective. No other dietary factors reached significance, suggesting dried fruits and cereals as potential modifiable protective factors against uterine inflammation.

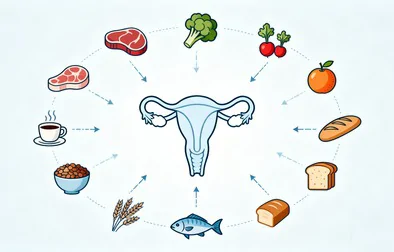

## 1 Introduction

Uterine inflammatory diseases, including endometritis and chronic endometritis, are characterized by localized inflammation of the endometrium and may lead to pelvic pain, infertility, and adverse reproductive outcomes such as recurrent pregnancy loss or implantation failure (Kimura et al. 2019; Park et al. 2016; Puente et al. 2020). The etiology of these diseases remains incompletely understood, with both infectious and non‐infectious factors implicated. Diet is a key modifiable determinant of inflammation, with specific nutrients and food groups demonstrating anti‐inflammatory or pro‐inflammatory effects (Sears 2015; Bolte et al. 2021; Galland 2010). Despite growing evidence linking diet to systemic inflammation, research on dietary factors in uterine inflammatory diseases remains limited, and their potential role in modulating endometrial inflammation is poorly understood. To address this gap, we performed a Mendelian randomization (MR) analysis investigating 19 dietary exposures from the UK Biobank, including: (1) animal‐based foods (processed meat, poultry, beef, pork, lamb/mutton, non‐oily fish, oily fish, cheese); (2) plant‐based foods (fresh fruit, dried fruit, cooked vegetables, salad/raw vegetables, bread, cereal); (3) beverages (alcohol intake frequency, tea, coffee, water intake); and (4) other dietary components (salt added to food), in relation to uterine inflammatory disease risk. MR offers a powerful alternative by leveraging genetic variants as instrumental variables to infer causal relationships (Ference et al. 2021; Grover et al. 2017). By using fixed genetic proxies for dietary exposures, MR minimizes confounding and avoids biases inherent in traditional observational designs.

## 2 Method

### 2.1 Source Data and Instrumental Variable Selection

For this MR study, genetic instruments for 19 dietary factors (e.g., alcohol intake, coffee consumption, fruit intake; full list in Table 1) were obtained from the UK Biobank (UKB) genome‐wide association studies (GWAS), ensuring robust and population‐level exposure data. The dietary factors involved in the study were measured using the ACE touchscreen questionnaire from the UKB, which mainly includes two categories: frequency and intake amount (Biobank 2025). The outcome, inflammatory disease of the uterus (ICD‐10: N71), was derived from FinnGen Biobank. Due to the use of data sourced from different databases, the overlap rate of our samples is nearly zero. The MR analysis relied on three key assumptions: (1) genetic variants strongly associate with the exposures, (2) variants are independent of confounders, and (3) they influence the outcome only via the exposure. To ensure robust and independent genetic instruments, we selected SNPs associated with dietary exposures at genome‐wide significance (*p* < 5 × 10^−8^), applied a 10,000 kb clumping window with a stringent LD threshold (*r* ^2^ < 0.001), and excluded any variants significantly linked to the outcome (*p* < 5 × 10^−8^) to minimize pleiotropic bias. This study utilized publicly available summary‐level GWAS data from UKB and FinnGen, which were previously approved by relevant ethics committees. Since no individual‐level data were accessed and all analyses were conducted in anonymized form, this research qualifies for exemption from additional institutional ethics review per applicable guidelines.

**TABLE 1:** Characteristics of GWAS datasets for dietary exposures and health outcomes.

ID	GWAS identifier	Variable	Sample size
1	ukb‐b‐5779	Alcohol intake frequency	462,346 individuals from the UK Biobank
2	ukb‐b‐6324	Processed meat intake	461,981 individuals from the UK Biobank
3	ukb‐b‐8006	Poultry intake	461,900 individuals from the UK Biobank
4	ukb‐b‐2862	Beef intake	461,053 individuals from the UK Biobank
5	ukb‐b‐17,627	Non‐oily fish intake	460,880 individuals from the UK Biobank
6	ukb‐b‐2209	Oily fish intake	460,443 individuals from the UK Biobank
7	ukb‐b‐5640	Pork intake	460,162 individuals from the UK Biobank
8	ukb‐b‐14,179	Lamb/mutton intake	460,006 individuals from the UK Biobank
9	ukb‐b‐11,348	Bread intake	452,236 individuals from the UK Biobank
10	ukb‐b‐1489	Cheese intake	451,486 individuals from the UK Biobank
11	ukb‐b‐8089	Cooked vegetable intake	448,651 individuals from the UK Biobank
12	ukb‐b‐6066	Tea intake	447,485 individuals from the UK Biobank
13	ukb‐b‐3881	Fresh fruit intake	446,462 individuals from the UK Biobank
14	ukb‐b‐15,926	Cereal intake	441,640 individuals from the UK Biobank
15	ukb‐b‐1996	Salad/raw vegetable intake	435,435 individuals from the UK Biobank
16	ukb‐b‐5237	Coffee intake	428,860 individuals from the UK Biobank
17	ukb‐b‐16,576	Dried fruit intake	421,764 individuals from the UK Biobank
18	ukb‐b‐8121	Salt added to food	462,630 individuals from the UK Biobank
19	ukb‐b‐14,898	Water intake	427,588 individuals from the UK Biobank
20	N14_INFLUTH	Inflammatory disease of uterus (ICD‐10: N71)	6060 cases and 250,564 controls from the FinnGen Release 12

*Note:* This table presents the characteristics of 20 GWAS datasets, including dietary exposures and health outcomes. The datasets primarily comprise UK Biobank participants, with sample sizes ranging from 421,764 to 462,630 individuals for dietary traits. The exception is the FinnGen dataset (ID 20), which includes 6060 cases and 250,564 controls for inflammatory disease of the uterus. Each entry lists the GWAS identifier, variable name, and corresponding sample details.

### 2.2 Statistical Analysis

We performed MR analyses using the TwoSampleMR package (version 0.6.2) in R (version 4.5.0). The primary analysis employed the inverse‐variance weighted (IVW) method, with *p* values corrected for multiple testing using the false discovery rate (FDR). Secondary analyses included the weighted median (WM) method and MR‐Egger regression. To evaluate potential violations of MR assumptions, we conducted several sensitivity analyses: (1) MR‐Egger regression was used to assess horizontal pleiotropy, with its intercept test indicating directional pleiotropy if statistically significant; (2) the MR‐PRESSO (Mendelian Randomization Pleiotropy RESidual Sum and Outlier) test was applied to detect and correct for potential outliers; and (3) Cochran's Q‐test was performed to examine heterogeneity across instrumental variable estimates.

## 3 Result

The complete analytical results, including effect estimates from multiple MR methods (IVW, MR‐Egger, and weighted median), sensitivity analyses (heterogeneity tests), and pleiotropy assessments, are presented in Table S1. Table 2 specifically summarizes the primary findings from the IVW method along with horizontal pleiotropy evaluation using the MR‐Egger intercept.

**TABLE 2:** Associations between dietary exposures and inflammatory disease of the uterus.

Serial number	Outcome	Exposure	Number of SNPs	IVW method					Horizontal pleiotropy		
				OR	lci95	uci95	*p* value	FDR‐corrected *p* value	Egger intercept	se	*p* value
1	Inflammatory disease of uterus	Alcohol intake frequency	86	1.07E+00	8.38E‐01	1.36E+00	5.93E‐01	7.89E‐01	−4.05E‐03	9.11E‐03	6.58E‐01
2	Inflammatory disease of uterus	Processed meat intake	21	7.42E‐01	4.04E‐01	1.36E+00	3.36E‐01	5.80E‐01	−1.23E‐02	2.26E‐02	5.93E‐01
3	Inflammatory disease of uterus	Poultry intake	7	2.76E+00	7.01E‐01	1.09E+01	1.47E‐01	3.98E‐01	9.95E‐02	2.27E‐01	6.80E‐01
4	Inflammatory disease of uterus	Beef intake	12	2.49E+00	9.20E‐01	6.72E+00	7.27E‐02	3.45E‐01	−2.56E‐02	3.69E‐02	5.04E‐01
5	Inflammatory disease of uterus	Non‐oily fish intake	11	8.09E‐01	2.65E‐01	2.47E+00	7.10E‐01	7.89E‐01	−1.83E‐02	3.42E‐02	6.07E‐01
6	Inflammatory disease of uterus	Oily fish intake	52	1.07E+00	7.16E‐01	1.59E+00	7.50E‐01	7.89E‐01	5.06E‐05	1.23E‐02	9.97E‐01
7	Inflammatory disease of uterus	Pork intake	10	2.36E+00	3.46E‐01	1.61E+01	3.81E‐01	6.03E‐01	−4.11E‐02	6.15E‐02	5.23E‐01
8	Inflammatory disease of uterus	Lamb/mutton intake	26	1.11E+00	5.14E‐01	2.41E+00	7.87E‐01	7.89E‐01	−2.09E‐03	1.83E‐02	9.10E‐01
9	Inflammatory disease of uterus	Bread intake	25	1.12E+00	6.27E‐01	2.01E+00	6.95E‐01	7.89E‐01	1.38E‐02	2.00E‐02	4.97E‐01
10	Inflammatory disease of uterus	Cheese intake	53	8.10E‐01	5.60E‐01	1.17E+00	2.64E‐01	5.41E‐01	−1.06E‐02	1.34E‐02	4.33E‐01
11	Inflammatory disease of uterus	Cooked vegetable intake	15	6.48E‐01	2.17E‐01	1.94E+00	4.37E‐01	6.39E‐01	1.09E‐02	6.69E‐02	8.74E‐01
12	Inflammatory disease of uterus	Tea intake	33	7.28E‐01	4.82E‐01	1.10E+00	1.32E‐01	3.98E‐01	−7.80E‐04	8.97E‐03	9.31E‐01
13	Inflammatory disease of uterus	Fresh fruit intake	49	5.04E‐01	2.44E‐01	1.04E+00	6.44E‐02	3.45E‐01	2.18E‐03	1.16E‐02	8.52E‐01
14	Inflammatory disease of uterus	Cereal intake	34	4.46E‐01	2.55E‐01	7.83E‐01	4.88E‐03	4.64E‐02	−9.65E‐03	1.72E‐02	5.78E‐01
15	Inflammatory disease of uterus	Salad/raw vegetable intake	12	3.32E‐01	8.29E‐02	1.33E+00	1.19E‐01	3.98E‐01	−4.28E‐02	3.29E‐02	2.23E‐01
16	Inflammatory disease of uterus	Coffee intake	35	9.33E‐01	5.63E‐01	1.55E+00	7.89E‐01	7.89E‐01	4.48E‐04	8.54E‐03	9.58E‐01
17	Inflammatory disease of uterus	Dried fruit intake	33	2.77E‐01	1.54E‐01	4.98E‐01	1.72E‐05	3.27E‐04	6.60E‐03	1.66E‐02	6.93E‐01
18	Inflammatory disease of uterus	Salt added to food	87	1.28E+00	8.92E‐01	1.83E+00	1.82E‐01	4.32E‐01	−6.52E‐03	8.57E‐03	4.49E‐01
19	Inflammatory disease of uterus	Water intake	33	7.65E‐01	4.68E‐01	1.25E+00	2.85E‐01	5.41E‐01	−2.95E‐03	1.04E‐02	7.79E‐01

*Note:* Odds ratios (ORs) with 95% confidence intervals (CIs) for the associations between various dietary exposures and inflammatory disease of the uterus, derived from Mendelian randomization analysis using the inverse‐variance weighted (IVW) method. Horizontal pleiotropy was assessed using the MR‐Egger intercept. FDR (false discovery rate)‐corrected *p* values account for multiple testing.

Abbreviations: lci95: lower 95% confidence interval; SNP: single nucleotide polymorphism; uci95: upper 95% confidence interval.

As shown in Table 2, the IVW analysis revealed statistically significant inverse associations for two dietary factors: dried fruit intake (OR = 0.277, 95% CI: 0.154–0.498, *p* = 1.72 × 10^−5^; FDR‐corrected *p* = 3.27 × 10^−4^) and cereal intake (OR = 0.446, 95% CI: 0.255–0.783, *p* = 4.88 × 10^−3^; FDR‐corrected *p* = 0.0464). These results suggest potential protective effects against inflammatory uterine disease. The WM analysis further supported the protective association of dried fruit intake (OR = 0.237, 95% CI: 0.107–0.526, *p* = 4.01 × 10^−4^), though cereal intake no longer reached statistical significance (OR = 0.525, 95% CI: 0.249–1.108, *p* = 0.0910). In contrast, the MR‐Egger method showed no significant associations for either dietary factor (dried fruit or cereal intake) with inflammatory uterine disease, suggesting that the protective effect of dried fruit intake was supported primarily by IVW and WM analyses. No other examined dietary exposures, including alcohol, meat products, fish, cheese, vegetables, fruits, or beverages, showed statistically significant associations (all nominal and FDR‐corrected *p* values > 0.05). Notably, MR‐Egger intercept tests detected no significant horizontal pleiotropy in any analysis (all intercept *p* values > 0.05). Detailed results from WM and MR‐Egger analyses are provided in Table S1.

## 4 Discussion

Our MR study identified dried fruit intake and cereal consumption as potential protective factors against inflammatory uterine diseases, with significant inverse associations in primary IVW analyses (OR = 0.277 and 0.446, respectively). These findings suggest that certain dietary components may mitigate uterine inflammation. The protective effect of dried fruits was further supported by the weighted median method, while cereal intake showed weaker consistency. No other dietary factors, including alcohol, meat, or fresh fruits, demonstrated significant causal relationships with inflammatory uterine diseases.

Diet plays a pivotal role as a modifiable risk factor influencing systemic inflammation (Christ et al. 2019). Scientific evidence demonstrates that particular food constituents can impact the production and regulation of hormones (Martin et al. 2019; Simó et al. 2015). Diet plays a critical role in shaping gut microbial diversity and the abundance of microbial metabolites, as seen in the stark contrasts between plant‐rich and protein‐rich dietary regimens (David et al. 2014). Alterations in gut microbiota composition can influence systemic estrogen levels—impacting uterine physiology—while also modulating local immune responses, altering gut metabolite profiles, and compromising intestinal barrier integrity (Bhat and Rao 2023). A study demonstrated that in lean women with polycystic ovary syndrome (PCOS), a high‐calorie breakfast with reduced dinner intake significantly decreased free testosterone and increased sex hormone‐binding globulin (SHBG) levels (Jakubowicz et al. 2013). Another cross‐sectional study (Liu et al. 2022) from the United States found that a pro‐inflammatory diet was associated with decreased SHBG levels. The interplay between SHBG and inflammation (e.g., CRP) has been widely documented in prior research (Liao et al. 2012; Azat Rasimovich and Rasim 2018). Diet affects metabolites in the blood, such as blood cell count, standard biochemistry, fatty acids, etc. (King et al. 2006; Hodson et al. 2008; Ambring et al. 2006), which may further influence uterine inflammation through systemic metabolic reprogramming. These pathways could partially explain the protective effects of certain dietary patterns observed in our study.

The robust inverse association observed for dried fruit intake (IVW OR = 0.277, *p* = 1.72 × 10^−5^) is biologically plausible. Dried fruits are rich in minerals, fiber, and micronutrients (e.g., potassium, folate, and vitamin C) with demonstrated anti‐inflammatory and antioxidant properties (Bennett et al. 2011). Dried fruits are a rich source of antioxidants that can scavenge free radicals such as superoxide, hydroxyl, and peroxyl radicals, and enhance plasma antioxidant capacity, thereby reducing oxidative damage at the cellular level (Wang et al. 2025, 2018; Alasalvar et al. 2020). For instance, a study demonstrated that daily consumption of 50–100 g dried plums by healthy postmenopausal women significantly increased total antioxidant capacity and superoxide dismutase activity over 6 months, while also lowering markers of oxidative stress and inflammation (Hong et al. 2021). Therefore, via these pathways, which include scavenging harmful free radicals, boosting systemic antioxidant capacity, and alleviating oxidative stress as well as inflammatory responses, dried fruits may reduce the oxidative burden on uterine tissues and thus potentially lower the risk of uterine inflammatory diseases. The consistency of this association in the weighted median analysis further strengthens its credibility, though the MR‐Egger results warrant caution (potential weak instrument bias or residual pleiotropy). In our study, the IVW analysis demonstrated an inverse association between cereal intake and the risk of uterine inflammatory diseases. Grains contain a composite of bioactive compounds, primarily including dietary fiber (e.g., arabinoxylan, β‐glucan) and polyphenols (predominantly phenolic acids like ferulic acid and vanillic acid), each of which contributes to robust anti‐inflammatory actions (Khan et al. 2024). These bioactive components in grains may serve as crucial pathways for reducing the risk of uterine inflammatory diseases. However, the absence of significant associations in both the WM and MR‐Egger analyses suggests that this relationship may lack robustness, potentially due to unmeasured confounders (e.g., variations in cereal processing methods or other dietary factors). Notably, no other dietary factors (e.g., alcohol, fresh fruits, or meat) showed significant associations. Both fruits and vegetables may exert anti‐inflammatory effects (in IVW method, the ORs for the associations between the intake of fresh fruits, dried fruits, fresh vegetables, and cooked vegetables and uterine inflammatory diseases are all less than 1). However, the results failed to reach statistical significance, which may be attributed to the relatively small number of cases of uterine inflammatory diseases (*n* = 6060). Our study provides compelling evidence that dried fruit intake and cereal consumption may serve as protective dietary factors against inflammatory uterine diseases. These findings carry important clinical implications for both preventive and therapeutic strategies in women's health.

Despite the clinically significant implications of our study, several limitations warrant consideration. First, although we employed multiple sensitivity analyses to address pleiotropy, residual confounding through unmeasured pathways (e.g., gut microbiome interactions) cannot be entirely excluded. Second, the broad categorization of exposures (e.g., ‘cereal intake’) prevented examination of potentially important subtypes (e.g., whole vs. refined grains). Third, our exclusive reliance on European‐ancestry populations may restrict the generalizability of findings to other ethnic groups with distinct genetic architectures or dietary patterns. Finally, the use of genetic proxies for lifetime exposure rather than measured dietary intake precluded assessment of critical windows for intervention or dose–response relationships that could refine clinical recommendations. To address the limitations of this study and extend its findings, future research should prioritize the following directions. First, the results should be replicated in non‐European populations to examine whether the protective associations of dried fruit and cereal intake against uterine inflammatory diseases are consistent across groups with different genetic backgrounds and cultural dietary patterns. This would enhance the generalizability of the findings. Second, more detailed dietary classification should be employed, for example, by distinguishing between unsweetened and sugar‐added dried fruits, as well as whole‐grain and refined cereals, to better capture nuanced dietary relationships. Third, validation of these associations through prospective studies, which reduce recall bias, and through interventional trials would help strengthen causal inference beyond the current associative evidence.

## 5 Conclusion

This study suggests that higher intake of dried fruits and cereals may reduce the risk of uterine inflammatory diseases, while other dietary factors showed no significant associations. The findings highlight the potential protective role of certain dietary patterns in women's health. Further research is needed to confirm these causal relationships and explore underlying biological mechanisms.

## Author Contributions

**Biao Xiong:** conceptualization (equal), data curation (equal), formal analysis (equal), funding acquisition (equal), investigation (equal), methodology (equal), project administration (equal), resources (equal), software (equal), supervision (equal), validation (equal), visualization (equal), writing – original draft (equal). **Zhaoan Lian:** conceptualization (equal), data curation (equal), formal analysis (equal), funding acquisition (equal), investigation (equal), methodology (equal), project administration (equal), resources (equal), software (equal), supervision (equal), validation (equal), visualization (equal), writing – original draft (equal).

## Conflicts of Interest

The authors declare no conflicts of interest.

## Supporting information


**Table S1:** Causal Relationships Between Dietary Exposures and inflammatory disease of uterus (ICD‐10: N71).
This Mendelian randomization study comprehensively evaluated the causal relationships between dietary factors and uterine inflammatory diseases. The analysis employed a robust methodological approach: (1) inverse‐variance weighted (IVW) regression served as the primary analytical method; (2) weighted median and MR‐Egger approaches provided supplementary causal estimates; (3) horizontal pleiotropy was assessed through MR‐Egger intercept tests (with statistical significance defined as *p* < 0.05); (4) heterogeneity was evaluated using Cochran's Q test; and (5) the MR‐PRESSO method identified and adjusted for potential outliers, with comparative results presented before and after outlier correction.

## Data Availability

All genetic association data used in this study are publicly available through the IEU Open GWAS database (https://gwas.mrcieu.ac.uk/) and FinnGen release R12 (https://www.finngen.fi/en).
