# Correction to “Causal Associations Between Dietary Factors and Uterine Inflammatory Diseases: A Mendelian Randomization Study”

**DOI:** 10.1002/fsn3.72146

**Published:** 2026-07-31

Xiong B. , and Lian Z. “Causal Associations Between Dietary Factors and Uterine Inflammatory Diseases: A Mendelian Randomization Study.” Food Science & Nutrition. 10.1002/fsn3.71076. Article number: e71076.PMC1254113341132577

The affiliation of the authors has been corrected from “Department of Obstetrics and Gynecology, Enshi Central Hospital, Enshi, Hubei, China” to “Department of Obstetrics and Gynecology, The Central Hospital of Enshi Tujia and Miao Autonomous Prefecture, Enshi, Hubei, China.”

The online version of this article has been updated accordingly.

We apologize for this error.

